# Human-specific mutations in VMAT1 confer functional changes and multi-directional evolution in the regulation of monoamine circuits

**DOI:** 10.1186/s12862-019-1543-8

**Published:** 2019-12-02

**Authors:** Daiki X. Sato, Yuu Ishii, Tomoaki Nagai, Kazumasa Ohashi, Masakado Kawata

**Affiliations:** Graduate School of Life Sciences, Tohoku University, Sendai, 980-8578 Japan

**Keywords:** VMAT1, *SLC18A1*, Human evolution, Monoamine transporter, Anxiety

## Abstract

**Background:**

Neurochemicals like serotonin and dopamine play crucial roles in human cognitive and emotional functions. Vesicular monoamine transporter 1 (VMAT1) transports monoamine neurotransmitters, and its variant (136Thr) is associated with various psychopathological symptoms and reduced monoamine uptake relative to 136Ile. We previously showed that two human-specific amino acid substitutions (Glu130Gly and Asn136Thr/Ile) of *VMAT1* were subject to positive natural selection. However, the potential functional alterations caused by these substitutions (Glu130Gly and Asn136Thr) remain unclear. To assess functional changes in *VMAT1* from an evolutionary perspective, we reconstructed ancestral residues and examined the role of these substitutions in monoamine uptake in vitro using fluorescent false neurotransmitters (FFN), which are newly developed substances used to quantitatively assay VMATs.

**Results:**

Immunoblotting confirmed that all the transfected YFP-VMAT1 variants are properly expressed in HEK293T cells at comparable levels, and no significant difference was seen in the density and the size of vesicles among them. Our fluorescent assays revealed a significant difference in FFN206 uptake among VMAT1 variants: 130Glu/136Asn, 130Glu/136Thr, and 130Gly/136Ile showed significantly higher levels of FFN206 uptake than 130Gly/136Asn and 130Gly/136Thr, indicating that both 130Glu and 136Ile led to increased neurotransmitter uptake, for which 136Thr and 136Asn were comparable by contrast.

**Conclusions:**

These findings suggest that monoamine uptake by VMAT1 initially declined (from 130Glu/136Asn to 130Gly/136Thr) in human evolution, possibly resulting in higher susceptibility to the external environment of our ancestors.

## Background

Understanding the nature of human behavior and its genetic underpinnings is a crucial issue in neuropsychology and evolutionary genetics. High cognitive and social abilities in humans, as represented by language, empathy, and altruism, are considered unique to our species. Brain neurochemicals such as serotonin and dopamine are known to underlie many important cognitive and emotional functions [1–4]. A recently proposed neurochemical hypothesis suggests that humans possess a dopamine-dominated striatum, i.e., a striatum with an elevated level of dopamine and a reduced level of acetylcholine that reflects our externally motivated behaviors [5–8]. Although monoamine neurotransmitters are produced in relatively few neurons in small areas of the brainstem, they are released widely and diffuse throughout the cerebral cortex [9]. Monoaminergic neurotransmitter systems have been implicated in various psychiatric disorders including depression, schizophrenia, attention-deficit hyperactivity disorder, and autism spectrum disorders [10–13]. As a result, regulatory monoaminergic pathways in the central nervous system have become targets for psychopharmacological interventions [14, 15].

The evolutionary origin of monoaminergic systems is thought to date back at least 600 million years to the stem metazoan (i.e., the common ancestor of Cnidaria and Bilateria) [16–19]. Social behavior regulated by monoaminergic systems is evolutionarily conserved in both vertebrates and invertebrates [20], suggesting that flexible behavioral control toward various environmental stimuli via monoamine neurotransmitters is advantageous for the survival of a wide range of taxa. Genetic differentiation in monoaminergic neuronal systems has been reported in several groups—especially among primates—and could be linked to the evolution of diverse emotional and/or social behaviors [21–28].

We previously showed that the vesicular monoamine transporter 1 (VMAT1; encoded by solute carrier family member A1 [*SLC18A1*]) was subject to positive selection in the human lineage [29]. *VMAT1* has evolved with two human-specific amino acid substitutions (from Glu to Gly at the 130th site and Asn to Thr at the 136th site). A new variant, namely 136Ile, emerged around the time of the Out-of-Africa (OoA) migration of modern humans and has achieved intermediate frequencies in non-African populations (20–61%). Since then, the Thr136Ile variant has been maintained through balancing selection in non-African populations [29]. To the best of our knowledge, 130Glu and 136Asn have not been reported in modern and/or archaic human populations.

The SLC18 family is a part of the major facilitator superfamily (MFS), the largest family of secondary active membrane transporters, whose members transport various substrates [30]. Within this family, VMATs are responsible for the accumulation of monoamines in synaptic vesicles. VMAT1 was initially thought to be expressed mainly in neurons of the peripheral nervous system and chromaffin cells, while the isoform, VMAT2, was thought to be expressed primarily in the brain [31, 32]. However, there is accumulating evidence that VMAT1 is also expressed in the brain where it plays important roles [33–35]. Genetic variants of *VMAT1* have been implicated in schizophrenia, bipolar disorders, autism, anxiety, depression, and neuroticism [34, 36–39], suggesting that VMAT1 plays an important role in the evolution of psychiatric disorders and emotional behavior. While variants in other genes involved in monoaminergic system are well studied (e.g., serotonin transporter [40, 41]; D4 dopamine receptor [42]; monoamine oxidase A [43]), genetic variants of *VMAT1* have only started to receive attention in recent years. Moreover, many studies have examined genetic variation in plasma membrane transporters (serotonin, noradrenaline, and dopamine transporters), which are involved in synaptic neurotransmitter reuptake and contribute to the duration of signaling. In contrast, VMATs can contribute to the magnitude of signaling and may be more closely linked to mechanisms regulating synaptic neurotransmitter release [44].

It is highly likely that the two human-specific amino acid substitutions (Glu to Gly at the 130th site and Asn to Thr or Ile at the 136th site) affect the monoamine uptake efficiency of VMAT1 as these sites belong to the first luminal loop domain, which is considered a putative receptor-like structure that is crucial for the transport of monoamines mediated by G-proteins [45, 46]. In fact, at one of the two sites (Thr136Ile polymorphism, rs1390938), 136Thr shows lower monoamine transport into presynaptic vesicles than 136Ile [44, 46], which could relate to higher levels of anxiety, neuroticism and/or psychiatric disorders in 136Thr variant carriers [34, 38, 39]. Taken together, these findings suggest that the monoamine uptake efficiency of VMAT1 significantly influences neurotic personality traits and psychiatric disorders.

Based on previous findings of a relationship between the positively selected 136Thr variant and greater anxiety, we hypothesize that the two human-specific substitutions of VMAT1 have led to more anxious and depressed human minds over the course of evolution from ancestral primates to modern humans, until the new “*hyperfunction allele*” (136Ile) [44] with less psychiatric phenotypes emerged. Additionally, Inoue-Murayama et al. (2001) speculated that anxiety has been favored throughout human evolution from interspecific comparison of serotonin transporter variants [23]. In this study, we assessed differences in neurotransmitter uptake among all *VMAT1* genotypes at the two sites (Glu130Gly and Asn136Thr/Ile) that possibly arose during the course of human evolution using recently developed fluorescent false neurotransmitters (FFNs) [47, 48] in vitro. FFNs are newly developed fluorescent substrates that target VMATs to visualize the neurotransmitters contained in synaptic vesicles [47]. FFN206 was further developed for the uptake assay to measure the activity of VMATs in cultured cells [48]. FFN206 is effectively uptaken into intracellular acidic vesicles in VMAT2-expressing HEK293 cells and the extent of its uptake can be quantified by fluorometric measurement [48]. In the present study, we conducted an FFN206 uptake assay to measure VMAT1 activity and compared the uptake among its variants. We predict that the human variant 130Gly/136Thr shows lower monoamine uptake than the ancestral non-human variant 130Glu/136Asn, which may support the hypothesis that higher anxiety and neuroticism were initially favored during human evolution.

## Results

**Fig. 1:**
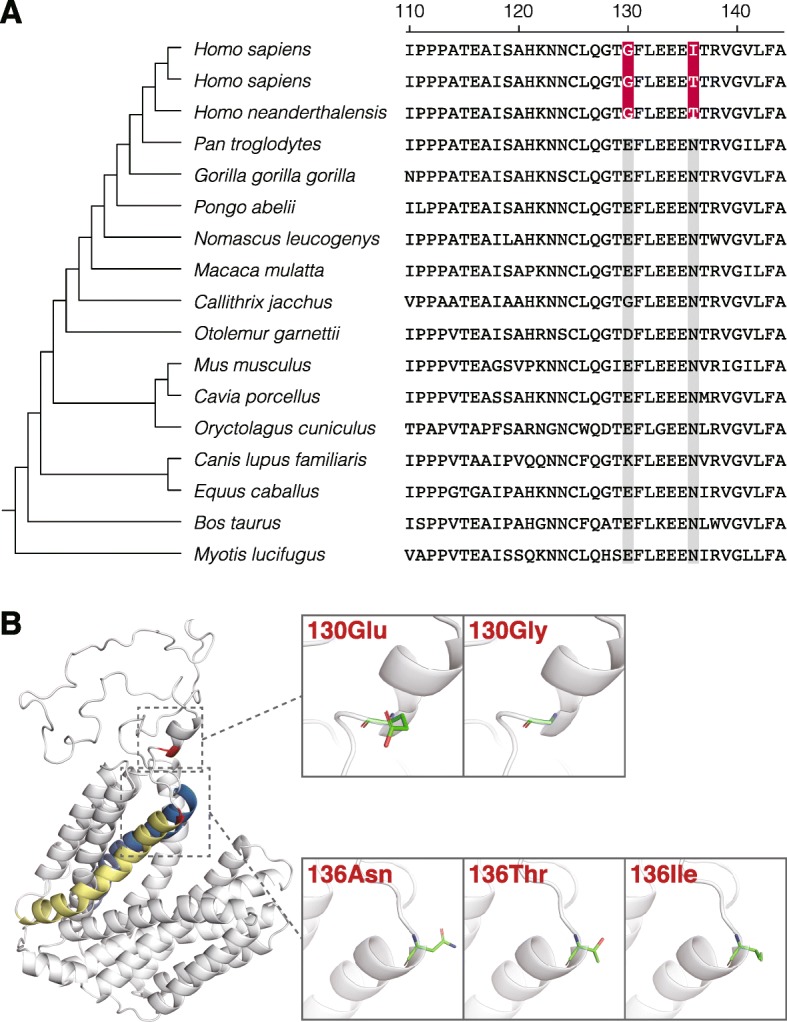
Amino acid sequence and protein structure of VMAT1. **a** Sequence alignments of VMAT1 among mammalian species show that the two conserved residues (highlighted in red) have changed specifically in humans. **b** Structure analysis shows that the 130th and 136th residues (highlighted in red) are located at the end of the loop domain between the first (highlighted in blue) and second (highlighted in yellow) transmembrane regions, as previously predicted. The side chains of the residues of interest are displayed in enlarged boxes

The sequence alignments and predicted structure of VMAT1 are shown in Fig. 1. Structure analysis showed that the 130th and 136th residues were located at the end of the loop domain between the first and second transmembrane regions (Fig. 1b), which is consistent with previous reports [44, 46].

**Fig. 2:**
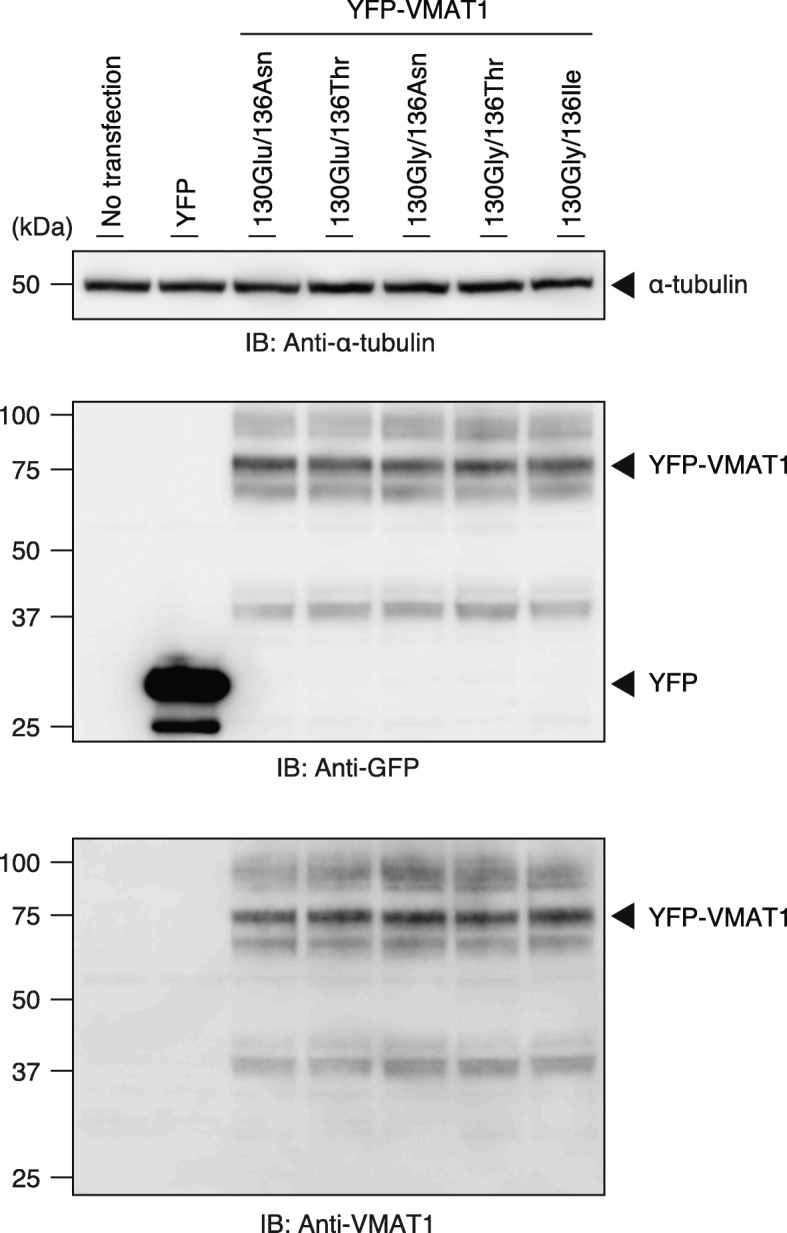
Western blotting for YFP-VMAT1 and its variants. Cells were transfected with plasmids encoding YFP, YFP-VMAT1, or its variants. Cells without transfection (no transfection) were also prepared (lane 1). Cell lysates were analyzed by immunoblotting with anti-GFP, anti-VMAT1, and anti-α-tubulin antibodies. Immunoblotting of α-tubulin is shown as a loading control. The major immunoreactive bands of GFP and VMAT1 at nearly 80 kDa are consistent with the putative molecular weight of the YFP-VMAT1 fusion protein. The full-length blots for all cropped ones are shown in (Additional file 1: Figure S1a)

To assess the efficiency of neurotransmitter uptake of the VMAT1 protein among its variants in human cells, we constructed an expression vector encoding YFP-tagged VMAT1 and generated a construct corresponding to each ancestral and derived genotype by site-directed mutagenesis. We carried out immunoblot analyses in HEK293T cell lysates and obtained the major immunoreactive bands of all YFP-VMAT1 variants at nearly 80 kDa, which is consistent with the putative molecular weight of the YFP-VMAT1 fusion protein (Fig. 2, Additional file 1: Figure S1a and b). We also obtained several upper bands of these lysates, likely due to post-translational glycosylation, as previously reported [49]. Densitometric analyses of YFP-VMAT1 immunoblots indicated that there was no significant difference in the level of protein expression among each YFP-VMAT1 variant (Additional file 1: Figure S1b and c). Taken together, these results confirm that all YFP-VMAT1 variants are properly expressed in HEK293T cells at comparable levels.

**Fig. 3:**
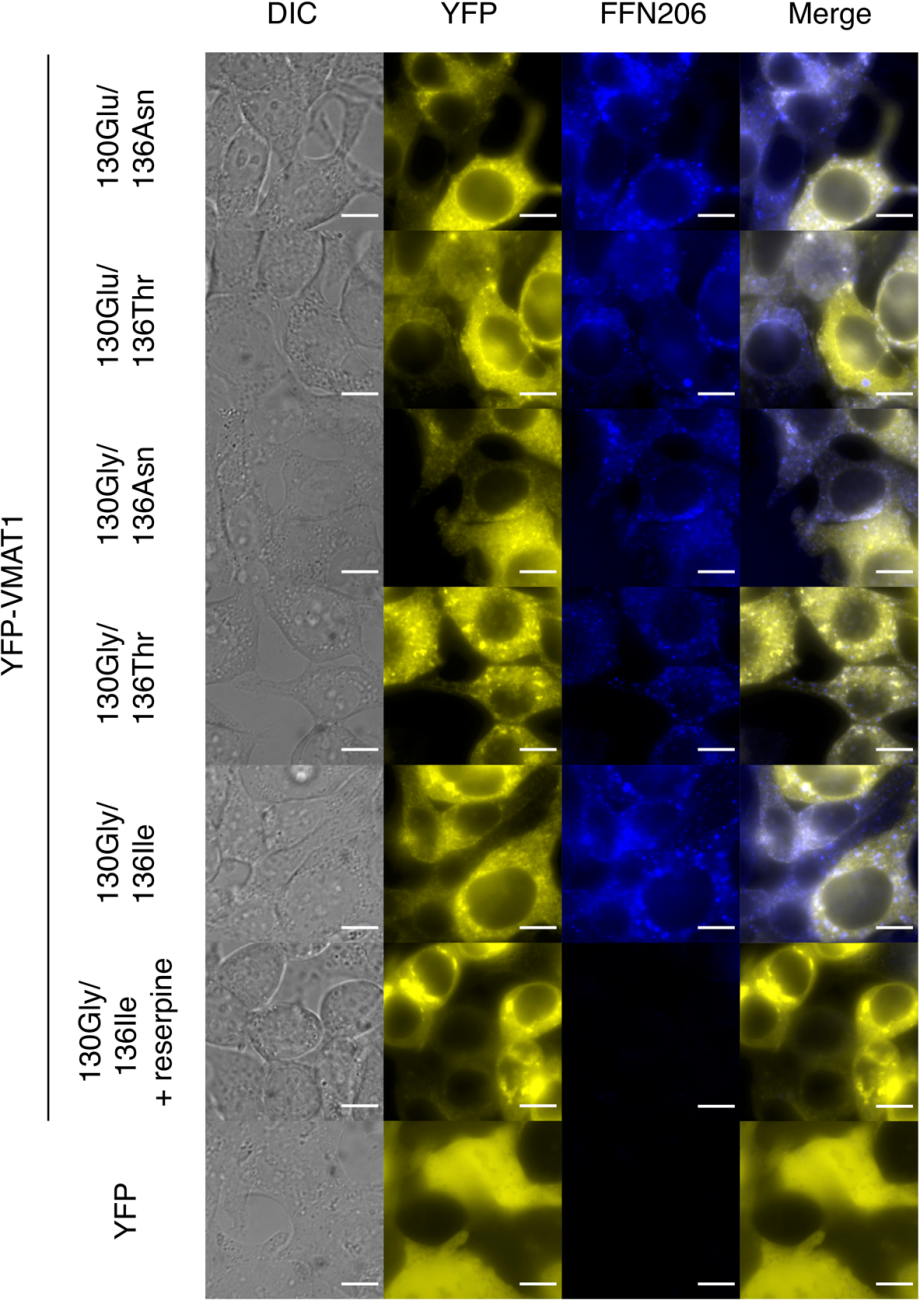
Fluorescence microscopy images of YFP-VMAT1-expressing HEK293T cells. Cells were transfected with plasmids encoding YFP, YFP-VMAT1, or its variants, pre-treated in the presence or absence of reserpine, and then incubated with FFN206. Cells were imaged by DIC (gray), the fluorescence of YFP (yellow), and FFN206 (blue). The right panels are merged images of YFP and FFN206 fluorescence. Scale bar = 10 μm

To investigate the effect of VMAT1 variants on neurotransmitter uptake, we performed FFN206 uptake assays in YFP-VMAT1-expressing HEK293T cells. HEK293T cells transfected with YFP or YFP-VMAT1 were incubated with FFN206, washed with PBS, and then imaged by YFP and FFN206 fluorescence (Fig. 3). We measured the fluorescence intensity of YFP and FFN206 and calculated the relative intensity of FFN206 by dividing its raw intensity by that of YFP to cancel the variation of YFP-VMAT1 expression in each cell.

**Fig. 4:**
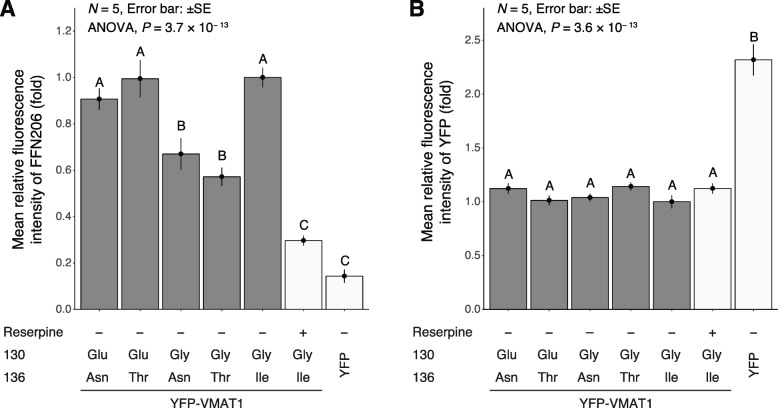
Expression level and functional activity of YFP-VMAT1 variants. **a** Quantification of uptake of FFN206 measured by the fluorescence intensity of FFN206 normalized to that of YFP and (**b**) expression level of each variant in each cell measured by the fluorescence intensity of YFP. The fluorescent intensity is shown in fold change to that of cells expressing YFP-VMAT1 130Gly/136Ile (without reserpine). Control conditions are shown in white bars. Data are the mean ± SEM from five independent experiments. In each experiment, 200 (lane 1–5) or 100 (lane 6 and 7) cells were measured. After observing significant differences among variants by one-way ANOVA (*P* = 3.7 × 10^− 13^ and *P* = 3.6 × 10^− 13^ for FFN206 uptake and expression level, respectively), analyses of variance with Tukey’s honestly significant difference (HSD) post-hoc tests were applied. Statistically significant differences (*P* < 0.05) are indicated by different letters. Detailed data are shown in (Additional file 2, 3, 4 and 5: Table S1–S4)

Our quantification showed that the level of FFN206 uptake was significantly lower in mock YFP-expressing cells than in any YFP-VMAT1-expressing cells, despite much higher expression of mock YFP protein (Figs. 3 and 4, lanes 1–5 and 7). Treatment with reserpine, a non-selective inhibitor for VMATs, also significantly reduced FFN206 uptake in cells expressing YFP-VMAT1 130Gly/136Ile without affecting the expression level of YFP-VMAT1 (Figs. 3 and 4, lanes 5 and 6). The results of these control experiments suggest that FFN206 uptake mainly reflects the intact activity of ectopically expressed YFP-VMAT1 and indicates the validity of our uptake assay for measuring VMAT1 activity.

Next, we compared the level of FFN206 uptake among cells expressing each YFP-VMAT1 variant. Strikingly, our quantification revealed a significant difference in FFN206 uptake among them: 130Glu/136Asn, 130Glu/136Thr, and 130Gly/136Ile showed significantly higher levels of FFN206 uptake than 130Gly/136Asn and 130Gly/136Thr (Figs. 3 and 4a, lanes 1–5). Detailed data and *P*-values calculated by Tukey’s honestly significant difference (HSD) are shown in (Additional files 2, 3, 4 and 5: Tables S1–S4). These results suggest that the amino acid substitutions at the 130th and 136th sites affect the neurotransmitter uptake of VMAT1.

To rule out the possibility that these differences in FFN206 fluorescence intensity was affected by the expression level and localization of YFP-VMAT1, we compared the YFP fluorescence intensity and the localization of YFP-VMAT1 among its variants in each cell. Quantification of YFP fluorescence showed that there were no significant differences in the levels of YFP intensity in each cell (Fig. 4b, lanes 1–5). We observed that all the YFP-VMAT1 variants exhibited vesicular localization in the cytoplasm (Fig. 3), and no significant difference was seen in the density and the size of vesicles among YFP-VMAT1 variants (Additional file 6: Figure S2), suggesting that the variation in FFN206 uptake was neither affected by the variation in expression level nor by the localization pattern of YFP-VMAT1 protein among its variants.

Our FFN206 uptake assay revealed the variation in VMAT1 function among its variants. Our comparison among variants indicated that both 130Glu and 136Ile led to increased neurotransmitter uptake, for which 136Thr and 136Asn were comparable by contrast (Figs. 3 and 4a, lanes 1–5).

## Discussion

Adopting an evolutionary perspective, the present study annotated the functional differences in neurotransmitter uptake of VMAT1 variants by fluorometric assay. The protein structure of VMAT1 showed that the amino acids at the 130th and 136th sites were located in the first luminal loop domain (Fig. 1b), which is known to be important for G-protein interactions that regulate VMAT1 activity [45]. This implies that the substitutions of these sites may affect the monoamine uptake efficiency of VMAT1. To address this issue, we performed the fluorometric assay measuring VMAT1-mediated uptake of fluorescent neurotransmitter FFN206. Critically, our assay revealed significant differences in the extent of FFN206 uptake among VMAT1 variants (Fig. 4a) without any significant difference in expression level or intracellular localization within each cell (Fig. 4b, Additional file 1: Figure S1 and Additional file 6: Figure S2). These results are compatible with the structural property that the amino acid substitutions are likely to affect functional activity. Our negative-control experiment showed that the expression of mock YFP scarcely increased the uptake of FFN206, and reserpine, a non-selective inhibitor for VMATs, strongly blocked it in VMAT1-expressing cells (Fig. 4a; lanes 6 and 7). These results suggest that the uptake of FFN206 depends on the activity of ectopically expressed YFP-VMAT1 variants and also indicates the validity of our uptake assay for measuring VMAT1 activity. The result that uptake of the 136Ile variant was higher than that of the 136Thr variant (Fig. 4a; lanes 4 and 5) is also consistent with that of the previous studies [44, 46]. In terms of the functional activity, 130Glu and 136Ile were associated with higher levels of FFN206 uptake, whereas no significant differences were observed between the 136Thr and 136Asn variants, which may reflect the biochemically similar properties of threonine and asparagine [50].

Given the evolutionary changes in amino acid sequence of *VMAT1* (Fig. 1a), those results indicate that the monoamine uptake by VMAT1 declined during the early phase of human evolution (i.e., from 130Glu/136Asn to 130Gly/136Thr) until the new variant 130Gly/136Ile emerged around the time of the OoA migration of modern humans [29]. The recently emerged 130Gly/136Ile should have conferred the elevated level of monoamine uptake by VMAT1, possibly linked to anti-psychotic brain circuits [38], and could have provided an evolutionary advantage to carriers against various environmental changes. Furthermore, our previous study indicated that 136Ile was under strong positive selective pressure in African populations where 136Ile is still at a low frequency [29]. It is of interest that non-existent ancestral variants of VMAT1 are functionally equivalent to existing variants (Fig. 4a; 130Glu/136Asn and 130Glu/136Thr to 130Gly/136Ile, and 130Gly/136Asn to 130Gly/136Thr). Considering the functional consequences, carriers of ancestral variants might exist in modern human populations. However, 130Glu or 136Asn have not yet been found in any publicly available human genome database, presumably due to a lack of the mutations or genetic drift that has eliminated these mutations.

The present study demonstrated that the variation of amino acids at the 130th and 136th of VMAT1 affected the uptake of fluorescent neurotransmitter. The Thr136Ile variant has been shown to affect Vmax (the maximum transport rate of monoamines) but not Km (the affinity of monoamines to transporters) [46]. Thus, the adjacent Glu130Gly variant could affect function in the same vein: amino acid changes could affect transport activity through a mechanism related not to binding but to dissociation with ligands [51]. Genetic changes in *VMAT1* could thus affect the amount of monoamines contained in vesicles, which is in turn released from the nerve terminal, affecting downstream signaling. A recent study has shown that *VMAT1* knockout in mice lead to differential dopamine signaling [52], and several other studies have revealed the association of the 136Thr with a variety of psychiatric phenotypes including alcohol dependence [38, 53], bipolar disorders [34], autism [39], anxiety [37, 38], maladaptive impulsivity, depressiveness, and neuroticism [38]. A functional neuroimaging study has revealed that this variant has distinct influences on activity of the prefrontal cortex and amygdala [44], both of which play crucial roles in emotional processing, that may underlie the socioemotional differences observed in the abovementioned studies.

The results presented here may support our hypothesis that evolutionary substitutions of two amino acids (from 130Glu/136Asn to 130Gly/136Thr) during early human evolution decreased monoamine uptake of VMAT1 and promoted increased levels of anxiety, depression, and neuroticism, given several lines of evidence for association of the 130Gly/136Thr variant with neuropsychiatric phenotypes. This is very likely in that we used to be exposed to high predation risks [54] and/or competition with conspecifics [55] in the ancestral environment, which possibly favors high levels of anxiety. Indeed, a simulation study suggested that risk sensitivity can evolve as an adaptive strategy in ancestral human populations [56]. However, it is difficult to infer the physiological and behavioral consequences of evolutionary changes in the VMAT1 protein in complicated epistatic networks and how it fits the neurological evidence on the evolutionary changes in neurochemical profiles in the human lineage [5, 6, 8]. To further conclude the phenotypic effects of the VMAT1 variants, it would be necessary to utilize animal models such as genome-edited mice and to observe the physiological or psychological phenotypes in addition to the transcriptional regulation of related genes.

## Conclusions

Taken together, our finding suggests that monoamine uptake by VMAT1 initially declined in early human evolution (from 130Glu/136Asn to 130Gly/136Thr) but increased along with the emergence of the 130Gly/136Ile variant around the time of the OoA dispersal of modern humans. The evolution of VMAT1 may have had significant neurophysiological influences on the human brain, resulting in cognitive, emotional, and behavioral characteristics unique to our species. Further neurological and behavioral experiments using model animals are necessary to evaluate the functional changes caused by human-specific mutations in VMAT1.

## Methods

### Sequence alignment, ancestral inference, and protein structure prediction

Phylogenetic analyses using 15 mammal species were carried out as previously described [29]. The ancestral residues were inferred by codeml in PAML software [57]. Homology modeling of human VMAT1 protein structure was performed using the SWISS-MODEL web server (http://swissmodel.expasy.org) [58]. The MFS transporter of *Escherichia coli* (PDB: 3wdo), which has the highest identity to human VMAT1 of the available structure models [59], was selected as the template (*E*-value: 7.6 × 10^− 37^; sequence similarity: 28.1%), and the QMEAN4 Z-score given by SWISS-MODEL was − 8.46. Visualization of the predicted 3D structure was performed by PyMOL 2.2 (DeLanoScientific, San Carlos, CA).

### Reagents and antibodies

FFN206 [48] (Abcam, Cambridge, MA, USA) was used as a fluorescent ligand of VMAT1. Although there are no available FFNs that have been designed specifically targeting human VMAT1, FFN206 has been proven useful for quantifying human VMAT2 activity because of the physiological property that its K_m_ is equivalent to that of dopamine, and it competes for uptake of serotonin [48]. It is generally known that VMAT1 and VMAT2 are almost functionally equivalent and share ligands [45], which supports the potential functionality of FFN206 for VMAT1. The mouse polyclonal antibody against α-tubulin (1:700, Sigma-Aldrich, St Louis, MO, USA) and rabbit polyclonal antibodies against VMAT1 (1:1000, ab168347, Abcam) and GFP (1:4000, Thermo Fisher Scientific, Waltham, MA, USA) were used as primary antibodies. The anti-mouse IgG HRP-linked antibody (1:600, Cell Signaling Technology, Beverly, MA, USA) and anti-rabbit IgG HRP-linked whole antibody from donkey (1:1000, GE Healthcare Bio-Sciences, Japan) were used as secondary antibodies.

### Plasmid construction and mutagenesis

The plasmid encoding YFP-VMAT1 was constructed by the following procedure. Artificially synthesized *VMAT1* cDNA (GENEWIZ, South Plainfield, NJ, USA) according to the coding sequence of NM_001135691.2 (475–2049) from NCBI was amplified by polymerase chain reaction with Tks Gflex DNA Polymerase (Takara Bio, Japan) and appropriate primers (see Additional file 7: Table S5) so that *Bgl*II and *EcoR*I sites were added on the edges of the sequence. The thermal cycling protocol was as follows: 94 °C for 1 min, followed by 30 cycles of 98 °C for 10 s, 65 °C for 15 s and 68 °C for 1 min. Amplified *VMAT1* cDNAs were digested with *Bgl*II and *EcoR*I (both from New England Biolabs Japan Inc., Japan), and *Bgl*II-*VMAT1*-*EcoR*I cDNAs were then ligated into pEYFP-C1 (Takara Bio) that had been digested with *Bgl*II and *EcoR*I.

Introduction of the point mutation in *VMAT1* was carried out using the QuickChange site-directed mutagenesis kit (Agilent, Santa Clara, CA, USA). The thermal cycling protocol was as follows: 95 °C for 30 s followed by 12 cycles of 95 °C for 30 s, 55 °C for 1 min, and 68 °C for 7 min. Mutated *VMAT1* plasmids were then treated with *Dpn*I and transformed into competent DH5α *E. coli* (TOYOBO, Japan). All plasmids that we constructed were cloned and fully sequenced by Sanger sequencing to confirm no undesired mutations. The primers used for the site-directed mutagenesis and the sequence confirmation are shown in (Additional file 7: Table S5).

### Cell culture and transfection

HEK293T cells were purchased from ATCC (#CRL-3216) and cultured in Dulbecco’s modified Eagle’s medium supplemented with 10% fetal calf serum. Transfection with plasmids was carried out using the FuGENE 6 Transfection Reagent (Promega, Madison, WI, USA) according to the manufacturer’s instructions.

### Immunoblotting

Immunoblotting was carried out to determine the approximate size of the analyzed proteins. HEK293T cells were transfected with plasmids encoding YFP, YFP-VMAT1, or its variants. Cells without transfection were also prepared as a negative control. Cells were washed two times in phosphate-buffered saline (PBS) and then lysed with 1× Laemmli lithium dodecyl sulfate sample buffer (Bio-Rad, Hercules, CA, USA) with 50 mM dithiothreitol for 30 min at 60 °C. Cell lysates were homogenized using QIAshredder (QIAGEN, Germany), centrifuged at 9100 g for 5 min at room temperature, and analyzed by SDS-polyacrylamide gel electrophoresis (PAGE) using 10–20% e-PAGEL (ATTO, Japan). Precision Plus protein standards (Bio-Rad) were used as a sizing ladder. Proteins separated on SDS-PAGE were transferred onto polyvinylidene difluoride membranes (ATTO). The membranes were blocked in 1× iBind Flex Solution (Thermo Fisher Scientific) at room temperature and probed with appropriate primary and secondary antibodies using an iBind Flex device (Thermo Fisher Scientific) for 3 h in total. Immunoreactive protein bands were visualized using EzWestLumi plus (ATTO).

### Fluorometric assay of VMAT1 protein and its variants

HEK293T cells were seeded on poly-L-lysine-coated 35 mm glass bottom dishes (Iwaki Glass, Japan) at a density of 1.5–2.0 × 10^5^ cells/dish and grown at 37 °C in 5% CO_2_. After 24 h, cells were transfected with 800 ng of each plasmid. Two days after transfection, the transfected HEK293T cells had reached 80–90% confluence. The uptake of VMAT1 substrate was carried out using FFN206 as follows. Briefly, cells were washed with PBS and then incubated in 1.0 ml of experimental media (Leibovitz’s L-15 Medium, Thermo Fisher Scientific) containing FFN206 at a final concentration of 5 μM at 37 °C in 5% CO_2_ for 2 h. After incubation, the cells were washed with PBS and maintained in fresh experimental media. For pharmacological inhibition of VMAT1, the cells were treated with 4 μM reserpine (Wako, Japan) at 37 °C in 5% CO_2_ for 1 h before uptake, while other cells were treated with DMSO vehicle. The details of the methods above followed by the previous study that showed the functionality of FFN206 [48]. Fluorescence images (for more than 100 cells per condition) were acquired using a DMI6000B fluorescence microscope (Leica, Germany) equipped with a PL Apo 63× oil-immersion objective lens (NA 1.3), a Chroma custom filter cube (ex = 500 ± 20 nm, em = 535 ± 30 nm for fluorescence of YFP and ex = 360 ± 40 nm, em = 470 ± 40 nm for fluorescence of FFN206), and a cooled charge-coupled device camera (Cool SNAP HQ; Roper Scientific, Netherlands) driven by LAS AF Imaging Software (Leica). All images were adjusted to the same contrast and brightness, and the mean fluorescence intensity in each cell area was measured using Fiji software (NIH, Bethesda, MD, USA). We measured the fluorescence intensity of YFP and FFN206 in more than 100 cells per condition, and their mean was used as the representative intensity of the given condition. Moreover, we quantified the intracellular density and size of vesicles expressing YFP-VMAT1 by tracking YFP fluorescence to evaluate the effects of variants on protein localization. The fluorescence intensity of FFN206 was corrected by that of YFP in each cell to account for the expression level of the given variant. The experiments were repeated five times independently. Statistical analyses were performed using R software.

## Supplementary information

**Additional file 1: Figure S1.** (a) Full-length blots for Fig. 2. (b) Four additional experiments of immunoblotting for YFP-VMAT1 with anti-VMAT1 and anti-α-tubulin antibodies. (c) Relative expression levels of each variant of VMAT1 corrected by α-tubulin based on immunoblotting. No significant differences were observed among variants in either comparison (*P*-values calculated by one-way ANOVA: 0.99).

**Additional file 2: Table S1.** Mean relative fluorescence intensity for FFN206 for each YFP-VMAT1 variant. The values were normalized to the mean value of the 130Gly/136Ile variant.

**Additional file 3: Table S2.** Tukey’s HSD correction for the difference of fluorescent intensity for FFN206 among YFP-VMAT1 variants.

**Additional file 4: Table S3.** Mean relative fluorescence intensity for YFP for each YFP-VMAT1 variant. The values were normalized to the mean value of the 130Gly/136Ile variant.

**Additional file 5: Table S4.** Tukey’s HSD correction for the differences in fluorescence intensity for YFP among YFP-VMAT1 variants.

**Additional file 6: Figure S2.** Mean relative (a) density and (b) size of vesicles expressing the YFP-VMAT1 variants. All values are shown as the fold change relative to that of the 130Gly/136Ile variant (without reserpine). No significant differences were observed among variants in either comparison (*P*-values calculated by one-way ANOVA: 0.39 and 0.54, respectively).

**Additional file 7: Table S5.** Primers used in the present study.

## Data Availability

All data generated or analyzed during this study are included in this published article and the Additional files.

## Abbreviations

Asn: Asparagine
FFN: Fluorescent false neurotransmitter
Glu: Glutamic acid
Gly: Glycine
HSD: Honestly significant difference
Ile: Isoleucine
MFS: Major facilitator superfamily
OoA: Out-of-Africa
PAGE: Polyacrylamide gel electrophoresis
PBS: Phosphate-buffered saline
*SLC18A1*: Solute carrier family member A1
Thr: Threonine
VMAT1: Vesicular monoamine transporter 1

## References

[1] Myhrer T (2003). Neurotransmitter systems involved in learning and memory in the rat: a meta-analysis based on studies of four behavioral tasks. Brain Res Rev.

[2] Roussos P, Giakoumaki SG, Bitsios P (2009). Cognitive and emotional processing in high novelty seeking associated with the L-DRD4 genotype. Neuropsychologia..

[3] Abu-Akel A, Shamay-Tsoory S (2011). Neuroanatomical and neurochemical bases of theory of mind. Neuropsychologia..

[4] Uzefovsky F, Shalev I, Israel S, Edelman S, Raz Y, Mankuta D (2015). Oxytocin receptor and vasopressin receptor 1a genes are respectively associated with emotional and cognitive empathy. Horm Behav.

[5] Raghanti MA, Edler MK, Stephenson AR, Wilson LJ, Hopkins WD, Ely JJ (2016). Human-specific increase of dopaminergic innervation in a striatal region associated with speech and language: a comparative analysis of the primate basal ganglia. J Comp Neurol.

[6] Sousa André M. M., Zhu Ying, Raghanti Mary Ann, Kitchen Robert R., Onorati Marco, Tebbenkamp Andrew T. N., Stutz Bernardo, Meyer Kyle A., Li Mingfeng, Kawasawa Yuka Imamura, Liu Fuchen, Perez Raquel Garcia, Mele Marta, Carvalho Tiago, Skarica Mario, Gulden Forrest O., Pletikos Mihovil, Shibata Akemi, Stephenson Alexa R., Edler Melissa K., Ely John J., Elsworth John D., Horvath Tamas L., Hof Patrick R., Hyde Thomas M., Kleinman Joel E., Weinberger Daniel R., Reimers Mark, Lifton Richard P., Mane Shrikant M., Noonan James P., State Matthew W., Lein Ed S., Knowles James A., Marques-Bonet Tomas, Sherwood Chet C., Gerstein Mark B., Sestan Nenad (2017). Molecular and cellular reorganization of neural circuits in the human lineage. Science.

[7] Stephenson AR, Edler MK, Erwin JM, Jacobs B, Hopkins WD, Hof PR (2017). Cholinergic innervation of the basal ganglia in humans and other anthropoid primates. J Comp Neurol.

[8] Raghanti MA, Edler MK, Stephenson AR, Munger EL, Jacobs B, Hof PR (2018). A neurochemical hypothesis for the origin of hominids. Proc Natl Acad Sci.

[9] Lövheim H (2012). A new three-dimensional model for emotions and monoamine neurotransmitters. Med Hypotheses.

[10] Kapur S, Remington G (1996). Serotonin-dopamine interaction and its relevance to schizophrenia. Am J Psychiatry.

[11] Stahl SM (2000). Blue genes and the monoamine hypothesis of depression. J Clin Psychiatry.

[12] Cohen IL, Liu X, Schutz C, White BN, Jenkins EC, Brown WT (2003). Association of autism severity with a monoamine oxidase a functional polymorphism. Clin Genet.

[13] Oades RD (2008). Dopamine-serotonin interactions in attention-deficit hyperactivity disorder (ADHD). Prog Brain Res.

[14] Iversen L (2000). Neurotransmitter transporters: fruitful targets for CNS drug discovery. Mol Psychiatry.

[15] Stahl SM. Stahl’s essential psychopharmacology. neuroscientific basis and practical application. 4th edi. Cambridge: Cambridge University Press; 2013.

[16] Hay-Schmidt A (2000). The evolution of the serotonergic nervous system. Proc Biol Sci.

[17] Caveney S, Cladman W, Verellen L, Donly C (2006). Ancestry of neuronal monoamine transporters in the Metazoa. J Exp Biol.

[18] Nickel M (2010). Evolutionary emergence of synaptic nervous systems: what can we learn from the non-synaptic, nerveless Porifera?. Invertebr Biol.

[19] Yamamoto K, Vernier P. The evolution of dopamine systems in chordates. Front Neuroanat. 2011;5:1–21. 10.3389/fnana.2011.00021 PMC3070214 21483723

[20] Edsinger Eric, Dölen Gül (2018). A Conserved Role for Serotonergic Neurotransmission in Mediating Social Behavior in Octopus. Current Biology.

[21] Lesch KP, Gross J, Wolozin BL, Murphy DL, Riederer P (1993). Extensive sequence divergence between the human and rat brain vesicular monoamine transporter: possible molecular basis for species differences in the susceptibility to MPP+. J Neural Transm.

[22] Kaplan JR, Phillips-Conroy J, Fontenot MB, Jolly CJ, Fairbanks LA, Mann JJ (1999). Cerebrospinal fluid monoaminergic metabolites differ in wild anubis and hybrid (Anubis hamadryas) baboons: possible relationships to life history and behavior. Neuropsychopharmacology..

[23] Inoue-Murayama M, Niimi Y, Takenaka O, Murayama Y, Miyoshi K, Shapiro CM (2001). Evolution of personality-related genes in primates.

[24] Wendland JR, Lesch KP, Newman TK, Timme A, Gachot-Neveu H, Thierry B (2006). Differential functional variability of serotonin transporter and monoamine oxidase a genes in macaque species displaying contrasting levels of aggression-related behavior. Behav Genet.

[25] Raghanti MA, Stimpson CD, Marcinkiewicz JL, Erwin JM, Hof PR, Sherwood CC (2008). Differences in cortical serotonergic innervation among humans, chimpanzees, and macaque monkeys: a comparative study. Cereb Cortex.

[26] Bergey Christina M., Phillips-Conroy Jane E., Disotell Todd R., Jolly Clifford J. (2016). Dopamine pathway is highly diverged in primate species that differ markedly in social behavior. Proceedings of the National Academy of Sciences.

[27] Stimpson CD, Barger N, Taglialatela JP, Gendron-Fitzpatrick A, Hof PR, Hopkins WD (2016). Differential serotonergic innervation of the amygdala in bonobos and chimpanzees. Soc Cogn Affect Neurosci.

[28] Rogers J (2018). The behavioral genetics of nonhuman primates: status and prospects. Am J Phys Anthropol.

[29] Sato DX, Kawata M (2018). Positive and balancing selection on SLC18A1 gene associated with psychiatric disorders and human-unique personality traits. Evol Lett.

[30] Saier MHJ, Beatty JT, Goffeau A, Harley KT, Heijne WHM, Huang S-C (1999). The major facilitator superfamily. J Mol Microbiol Biotechnol.

[31] Peter D, Finn JP, Klisak I, Liu Y, Kojis T, Heinzmann C (1993). Chromosomal localization of the human vesicular amine transporter genes. Genomics..

[32] Erickson JD, Schäfer MK-H, Bonner TI, Eiden LE, Weihe E (1996). Distinct pharmacological properties and distribution in neurons and endocrine cells of two isoforms of the human vesicular monoamine transporter. Proc Natl Acad Sci U S A.

[33] Hansson SR, Hoffman BJ (1998). Mezey É́. Ontogeny of vesicular monoamine transporter mRNAs VMAT1 and VMAT2. I. the developing rat central nervous system. Dev Brain Res.

[34] Lohoff FW, Dahl JP, Ferraro TN, Arnold SE, Gallinat J, Sander T (2006). Variations in the vesicular monoamine transporter 1 gene (VMAT1/SLC18A1) are associated with bipolar I disorder. Neuropsychopharmacology..

[35] Multani PK (2013). Hodge R, Estévez M a., Abel T, kung H, Alter M, et al. VMAT1 deletion causes neuronal loss in the hippocampus and neurocognitive deficits in spatial discrimination. Neuroscience..

[36] Lohoff FW, Weller AE, Bloch PJ, Buono RJ, Doyle GA, Ferraro TN (2008). Association between polymorphisms in the vesicular monoamine transporter 1 gene (VMAT1/SLC18A1) on chromosome 8p and schizophrenia. Neuropsychobiology..

[37] Lohoff FW, Lautenschlager M, Mohr J, Ferraro TN, Sander T, Gallinat J (2008). Association between variation in the vesicular monoamine transporter 1 gene on chromosome 8p and anxiety-related personality traits. Neurosci Lett.

[38] Vaht M, Kiive E, Veidebaum T, Harro J (2016). A functional vesicular monoamine transporter 1 (VMAT1) gene variant is associated with affect and the prevalence of anxiety, affective, and alcohol use disorders in a longitudinal population-representative birth cohort study. Int J Neuropsychopharmacol.

[39] Noroozi R, Ghafouri-Fard S, Omrani MD, Habibi M, Sayad A, Taheri M (2017). Association study of the vesicular monoamine transporter 1 (VMAT1) gene with autism in an Iranian population. Gene..

[40] Lesch K.-P., Bengel D., Heils A., Sabol S. Z., Greenberg B. D., Petri S., Benjamin J., Muller C. R., Hamer D. H., Murphy D. L. (1996). Association of Anxiety-Related Traits with a Polymorphism in the Serotonin Transporter Gene Regulatory Region. Science.

[41] Hariri AR, Holmes A (2006). Genetics of emotional regulation: the role of the serotonin transporter in neural function. Trends Cogn Sci.

[42] Ebstein RP, Novick O, Umansky R, Priel B, Osher Y, Blaine D (1996). Dopamine D4 receptor (D4DR) exon III polymorphism associated with the human personality trait of novelty seeking. Nat Genet.

[43] Sabol SZ, Hu S, Hamer D (1998). A functional polymorphism in the monoamine oxidase a gene promoter. Hum Genet.

[44] Lohoff FW, Hodge R, Narasimhan S, Nall A, Ferraro TN, Mickey BJ (2012). Functional genetic variants in the vesicular monoamine transporter 1 modulate emotion processing. Mol Psychiatry.

[45] Brunk I, Blex C, Rachakonda S, Höltje M, Winter S, Pahner I (2006). The first luminal domain of vesicular monoamine transporters mediates G-protein-dependent regulation of transmitter uptake. J Biol Chem.

[46] Khalifa AM, Watson-Siriboe A, Shukry SG, Chiu WL, Nelson ME, Geng Y (2012). Thr136Ile polymorphism of human vesicular monoamine transporter-1 (SLC18A1 gene) influences its transport activity in vitro. Neuroendocrinol Lett.

[47] Gubernator NG, Zhang H, Staal RGW, Mosharov EV, Pereira DB, Yue M (2009). Fluorescent false neurotransmitters visualize dopamine release from individual presynaptic terminals. Science..

[48] Hu G, Henke A, Karpowicz RJ, Sonders MS, Farrimond F, Edwards R (2013). New fluorescent substrate enables quantitative and high-throughput examination of vesicular monoamine transporter 2 (VMAT2). ACS Chem Biol.

[49] Liu Y, Schweitzer ES, Nirenberg MJ, Pickel VM, Evans CJ, Edwards RH (1994). Preferential localization of a vesicular monoamine transporter to dense core vesicles in PC12 cells. J Cell Biol.

[50] Betts MJ, Russel RB (2003). Amino acid properties and consequences of substitutions. Bioinformatics for Geneticists.

[51] Parsons SM (2000). Transport mechanisms in acetylcholine and monoamine storage. FASEB J.

[52] Lohoff FW, Carr GV, Brookshire B, Ferraro TN, Lucki I (1712). Deletion of the vesicular monoamine transporter 1 (vmat1/slc18a1) gene affects dopamine signaling. Brain Res.

[53] Dutta N, Helton SG, Schwandt M, Zhu X, Momenan R, Lohoff FW (2016). Genetic variation in the vesicular monoamine transporter 1 (VMAT1/SLC18A1) gene and alcohol withdrawal severity. Alcohol Clin Exp Res.

[54] Turner JH (2014). The evolution of human emotions. Handbook of the sociology of emotions.

[55] Neuberg SL, Kenrick DT, Schaller M (2011). Human threat management systems: self-protection and disease avoidance. Neurosci Biobehav Rev.

[56] Hintze A, Olson RS, Adami C, Hertwig R (2015). Risk sensitivity as an evolutionary adaptation. Sci Rep.

[57] Yang Z (2007). PAML 4: phylogenetic analysis by maximum likelihood. Mol Biol Evol.

[58] Waterhouse A, Bertoni M, Bienert S, Studer G, Tauriello G, Gumienny R (2018). SWISS-MODEL: homology modelling of protein structures and complexes. Nucleic Acids Res.

[59] Yaffe Dana, Forrest Lucy R., Schuldiner Shimon (2018). The ins and outs of vesicular monoamine transporters. The Journal of General Physiology.

