# Supplementary figures and images for: Human-specific mutations in VMAT1 confer functional changes and multi-directional evolution in the regulation of monoamine circuits
Source: BMC Evol Biol. 2019 Dec 2;19:220. doi: 10.1186/s12862-019-1543-8 (PMC6889191; doi:10.1186/s12862-019-1543-8)

(a)

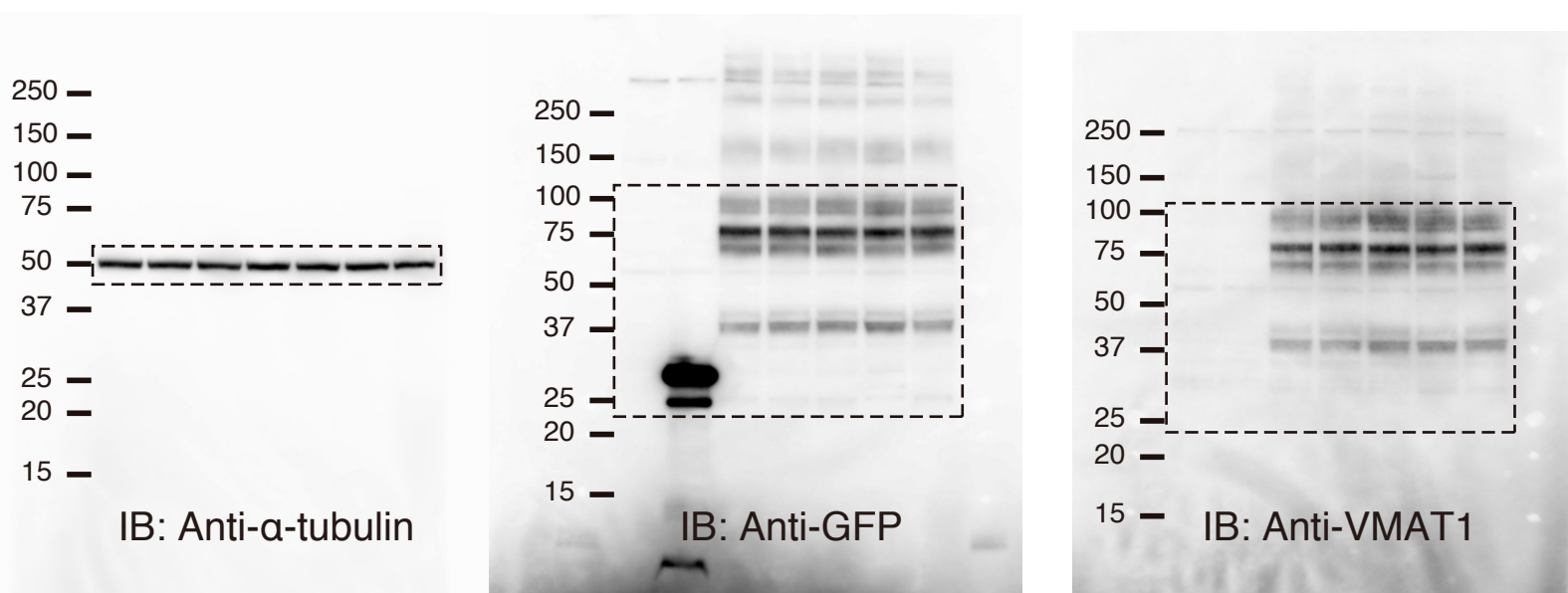

(b)

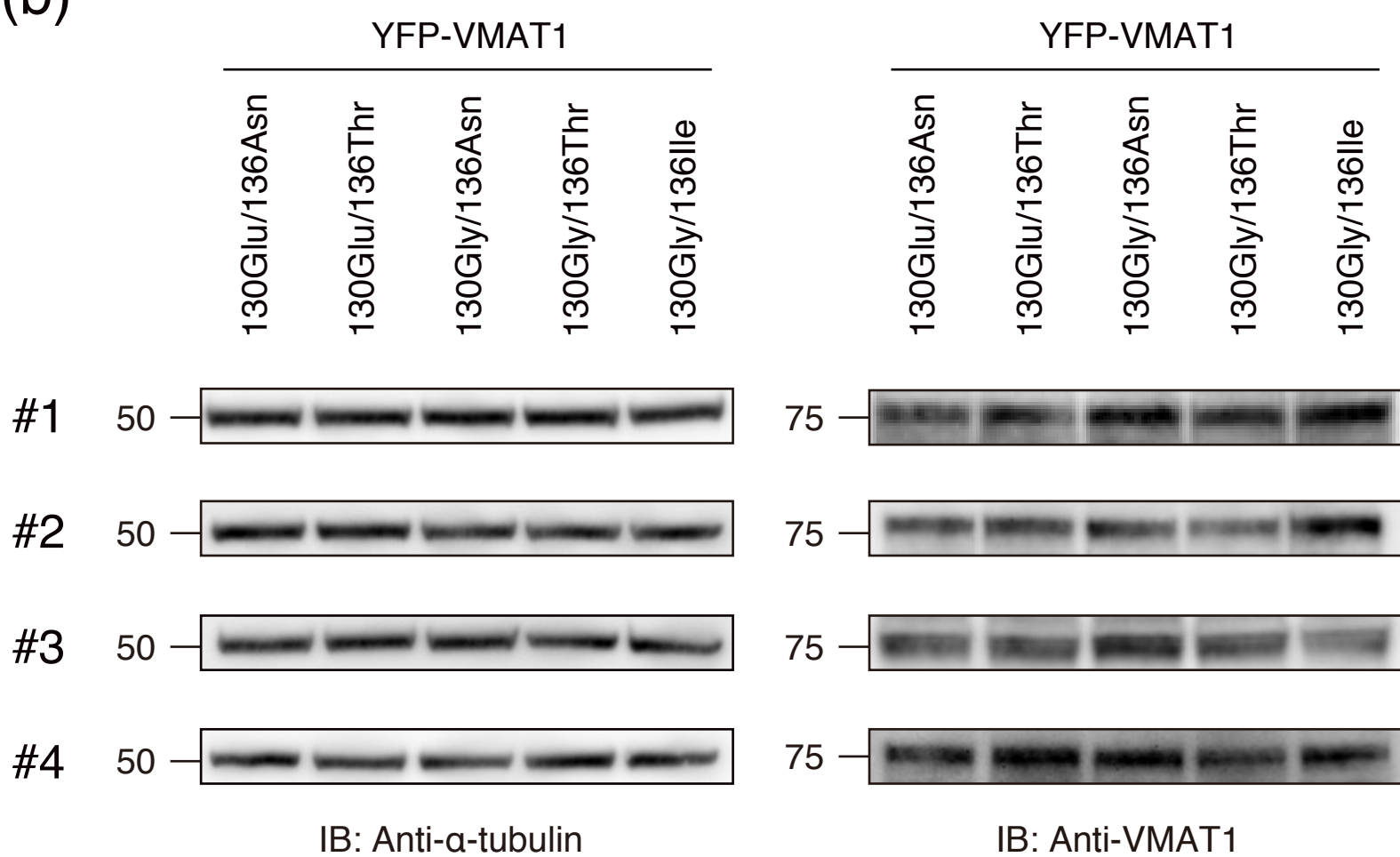

(c)

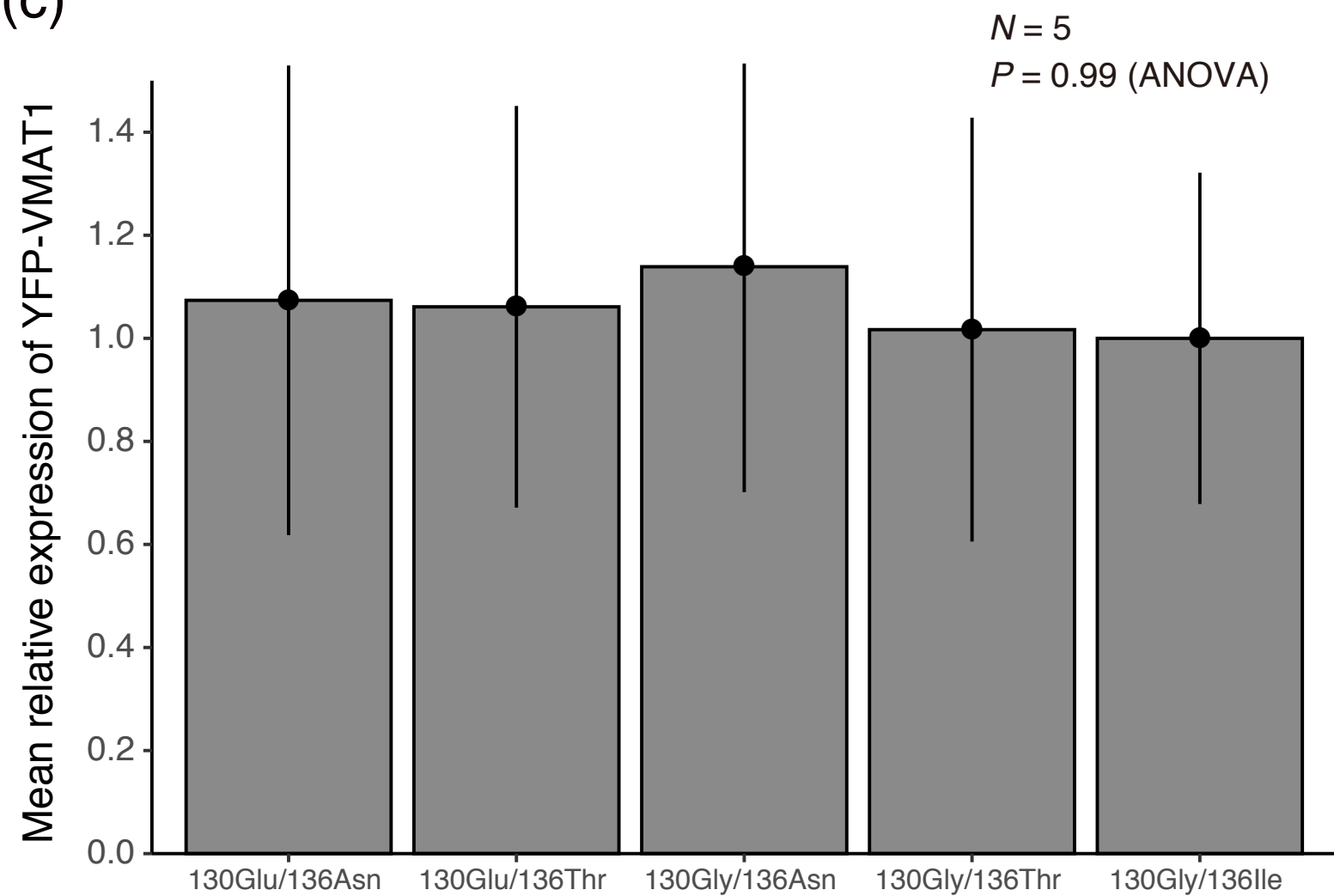

Supplement: Supplementary file 1 — Additional file 1: Figure S1. (a) Full-length blots for Fig. 2. (b) Four additional experiments of immunoblotting for YFP-VMAT1 with anti-VMAT1 and anti-α-tubulin antibodies. (c) Relative expression levels of each variant of VMAT1 corrected by α-tubulin based on immunoblotting. No significant differences were observed among variants in either comparison (P-values calculated by one-way ANOVA: 0.99). [file 12862_2019_1543_MOESM1_ESM.pdf]

(a) Vesicle density

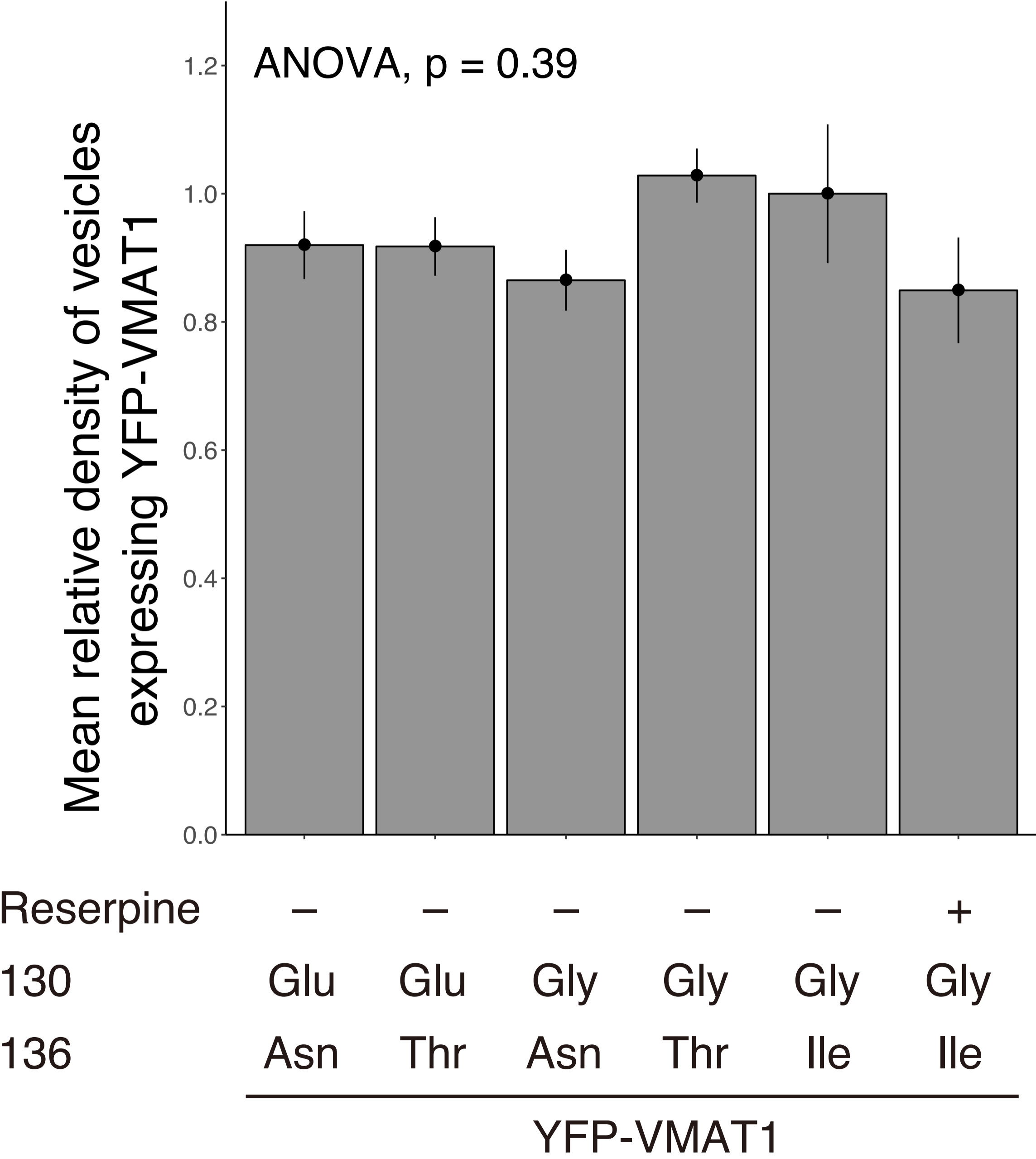

(b) Vesicle size

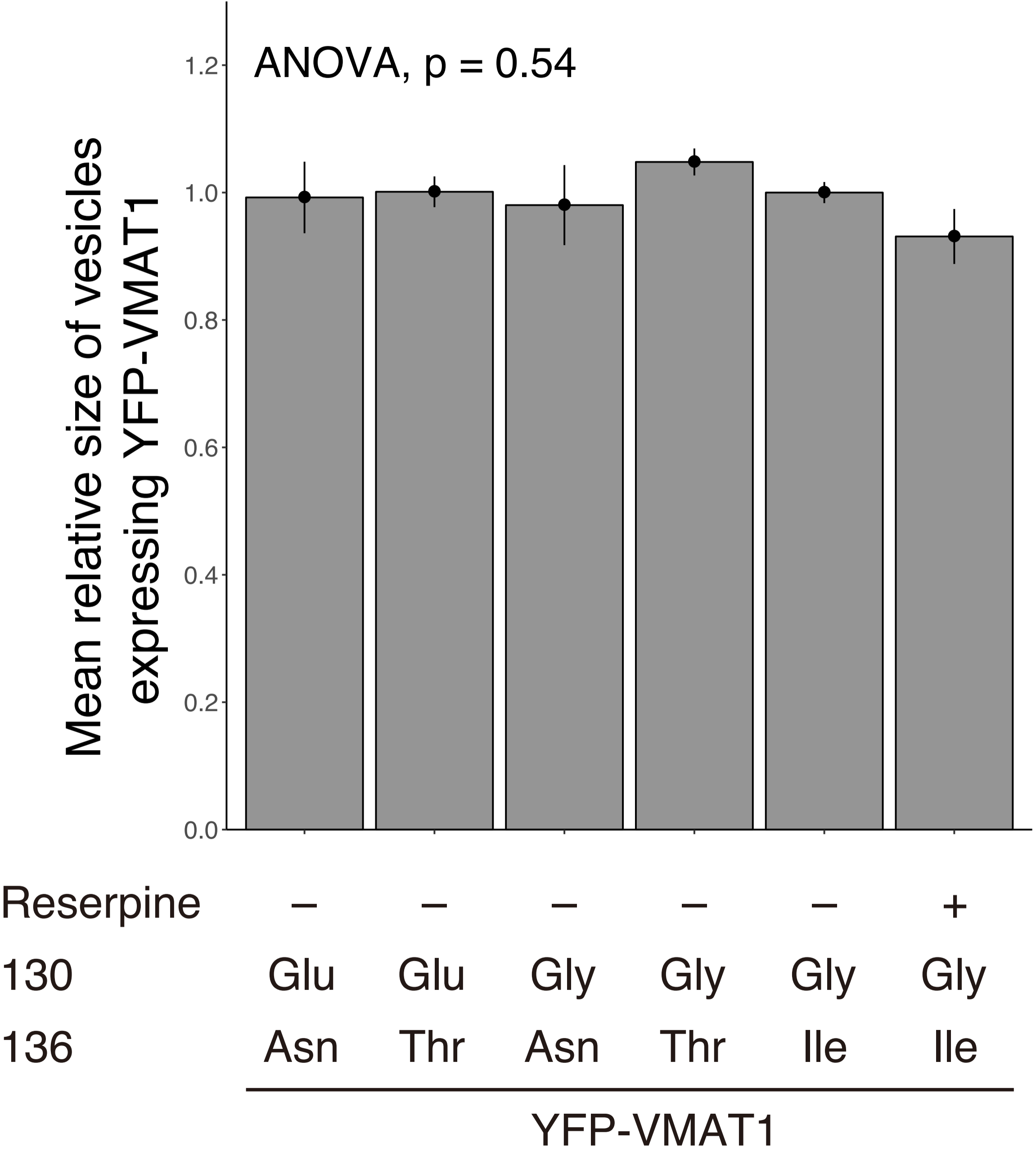

Supplement: Supplementary file 6 — Additional file 6: Figure S2. Mean relative (a) density and (b) size of vesicles expressing the YFP-VMAT1 variants. All values are shown as the fold change relative to that of the 130Gly/136Ile variant (without reserpine). No significant differences were observed among variants in either comparison (P-values calculated by one-way ANOVA: 0.39 and 0.54, respectively). [file 12862_2019_1543_MOESM6_ESM.pdf]
