# Supplementary material for: Human-specific mutations in VMAT1 confer functional changes and multi-directional evolution in the regulation of monoamine circuits
Source: BMC Evol Biol. 2019 Dec 2;19:220. doi: 10.1186/s12862-019-1543-8 (PMC6889191; doi:10.1186/s12862-019-1543-8)
Supplement: Supplementary file 2 — Additional file 2: Table S1. Mean relative fluorescence intensity for FFN206 for each YFP-VMAT1 variant. The values were normalized to the mean value of the 130Gly/136Ile variant. [file 12862_2019_1543_MOESM2_ESM.docx]

**Table S1**. Mean relative fluorescence intensity for FFN206 for each YFP-VMAT1 variant.

|  | 2018/6/25 | 2018/6/28 | 2018/8/6 | 2018/8/9 | 2018/8/13 |
| --- | --- | --- | --- | --- | --- |
| 130Glu/136Asn | 0.7487 | 0.9784 | 0.8562 | 0.9591 | 0.9918 |
| 130Glu/136Thr | 1.2277 | 0.9582 | 0.7428 | 0.9621 | 1.0823 |
| 130Gly/136Asn | 0.5763 | 0.8550 | 0.4949 | 0.6239 | 0.8010 |
| 130Gly/136Thr | 0.5068 | 0.6759 | 0.4627 | 0.5715 | 0.6423 |
| 130Gly/136Ile | 1.0710 | 1.0921 | 1.0345 | 0.8728 | 0.9296 |
| 130Gly/136Ile + reserpine | 0.3326 | 0.3080 | 0.2233 | 0.3440 | 0.2763 |
| YFP | 0.1933 | 0.1017 | 0.0977 | 0.2310 | 0.0931 |
