# Supplementary material for: Human-specific mutations in VMAT1 confer functional changes and multi-directional evolution in the regulation of monoamine circuits
Source: BMC Evol Biol. 2019 Dec 2;19:220. doi: 10.1186/s12862-019-1543-8 (PMC6889191; doi:10.1186/s12862-019-1543-8)
Supplement: Supplementary file 3 — Additional file 3: Table S2. Tukey’s HSD correction for the difference of fluorescent intensity for FFN206 among YFP-VMAT1 variants. [file 12862_2019_1543_MOESM3_ESM.docx]

**Table S2**. Tukey's HSD correction for the difference of fluorescent intensity for FFN206 among YFP-VMAT1 variants.

| Genotype–Genotype | difference | adjusted p-values |
| --- | --- | --- |
| 130Glu/136Asn–130Glu/136Thr | 0.0878 | 0.8768 |
| 130Glu/136Asn–130Gly/136Asn | -0.2366 | 0.0363 |
| 130Glu/136Asn–130Gly/136Thr | -0.3350 | 0.0011 |
| 130Glu/136Asn–130Gly/136Ile | 0.0932 | 0.8441 |
| 130Glu/136Asn–130Gly/136Ile + reserpine | -0.6100 | 0.0000 |
| 130Glu/136Asn–YFP | -0.7635 | 0.0000 |
| 130Glu/136Thr–130Gly/136Asn | -0.3244 | 0.0017 |
| 130Glu/136Thr–130Gly/136Thr | -0.4228 | 0.0000 |
| 130Glu/136Thr–130Gly/136Ile | 0.0054 | 1.0000 |
| 130Glu/136Thr–130Gly/136Ile + reserpine | -0.6978 | 0.0000 |
| 130Glu/136Thr–YFP | -0.8512 | 0.0000 |
| 130Gly/136Asn–130Gly/136Thr | -0.0984 | 0.8087 |
| 130Gly/136Asn–130Gly/136Ile | 0.3297 | 0.0014 |
| 130Gly/136Asn–130Gly/136Ile + reserpine | -0.3734 | 0.0003 |
| 130Gly/136Asn–YFP | -0.5269 | 0.0000 |
| 130Gly/136Thr–130Gly/136Ile | 0.4281 | 0.0000 |
| 130Gly/136Thr–130Gly/136Ile + reserpine | -0.2750 | 0.0099 |
| 130Gly/136Thr–YFP | -0.4285 | 0.0000 |
| 130Gly/136Ile–130Gly/136Ile + reserpine | -0.7032 | 0.0000 |
| 130Gly/136Ile–YFP | -0.8566 | 0.0000 |
| 130Gly/136Ile + reserpine–YFP | -0.1535 | 0.3537 |
