# Supplementary material for: Human-specific mutations in VMAT1 confer functional changes and multi-directional evolution in the regulation of monoamine circuits
Source: BMC Evol Biol. 2019 Dec 2;19:220. doi: 10.1186/s12862-019-1543-8 (PMC6889191; doi:10.1186/s12862-019-1543-8)
Supplement: Supplementary file 4 — Additional file 4: Table S3. Mean relative fluorescence intensity for YFP for each YFP-VMAT1 variant. The values were normalized to the mean value of the 130Gly/136Ile variant. [file 12862_2019_1543_MOESM4_ESM.docx]

**Table S3**. Mean relative fluorescence intensity for YFP for each YFP-VMAT1 variant.

|  | 2018/6/25 | 2018/6/28 | 2018/8/6 | 2018/8/9 | 2018/8/13 |
| --- | --- | --- | --- | --- | --- |
| 130Glu/136Asn | 1.0598 | 1.1572 | 1.0646 | 1.0279 | 1.3024 |
| 130Glu/136Thr | 1.0338 | 0.9298 | 1.0838 | 1.1261 | 0.8909 |
| 130Gly/136Asn | 0.9993 | 0.9866 | 1.1475 | 1.1018 | 0.9592 |
| 130Gly/136Thr | 1.2409 | 1.1760 | 1.1787 | 1.0988 | 1.0129 |
| 130Gly/136Ile | 1.1119 | 0.8845 | 0.9875 | 1.1647 | 0.8513 |
| 130Gly/136Ile + reserpine | 1.1533 | 1.0704 | 1.1312 | 1.2773 | 0.9821 |
| YFP | 2.5787 | 2.5737 | 2.3628 | 2.2895 | 1.7846 |
