# Supplementary material for: Human-specific mutations in VMAT1 confer functional changes and multi-directional evolution in the regulation of monoamine circuits
Source: BMC Evol Biol. 2019 Dec 2;19:220. doi: 10.1186/s12862-019-1543-8 (PMC6889191; doi:10.1186/s12862-019-1543-8)
Supplement: Supplementary file 5 — Additional file 5: Table S4. Tukey’s HSD correction for the differences in fluorescence intensity for YFP among YFP-VMAT1 variants. [file 12862_2019_1543_MOESM5_ESM.docx]

**Table S4**. Tukey's HSD correction for the differences in fluorescence intensity for YFP among YFP-VMAT1 variants.

| Genotype–Genotype | difference | adjusted p-values |
| --- | --- | --- |
| 130Glu/136Asn–130Glu/136Thr | -0.1095 | 0.9220 |
| 130Glu/136Asn–130Gly/136Asn | -0.0835 | 0.9782 |
| 130Glu/136Asn–130Gly/136Thr | 0.0191 | 1.0000 |
| 130Glu/136Asn–130Gly/136Ile | -0.1224 | 0.8755 |
| 130Glu/136Asn–130Gly/136Ile + reserpine | 0.0005 | 1.0000 |
| 130Glu/136Asn–YFP | 1.1955 | 0.0000 |
| 130Glu/136Thr–130Gly/136Asn | 0.0260 | 1.0000 |
| 130Glu/136Thr–130Gly/136Thr | 0.1286 | 0.8485 |
| 130Glu/136Thr–130Gly/136Ile | -0.0129 | 1.0000 |
| 130Glu/136Thr–130Gly/136Ile + reserpine | 0.1100 | 0.9205 |
| 130Glu/136Thr–YFP | 1.3050 | 0.0000 |
| 130Gly/136Asn–130Gly/136Thr | 0.1026 | 0.9417 |
| 130Gly/136Asn–130Gly/136Ile | -0.0389 | 0.9997 |
| 130Gly/136Asn–130Gly/136Ile + reserpine | 0.0840 | 0.9776 |
| 130Gly/136Asn–YFP | 1.2790 | 0.0000 |
| 130Gly/136Thr–130Gly/136Ile | -0.1415 | 0.7842 |
| 130Gly/136Thr–130Gly/136Ile + reserpine | -0.0186 | 1.0000 |
| 130Gly/136Thr–YFP | 1.1764 | 0.0000 |
| 130Gly/136Ile–130Gly/136Ile + reserpine | 0.1229 | 0.8735 |
| 130Gly/136Ile–YFP | 1.3179 | 0.0000 |
| 130Gly/136Ile + reserpine–YFP | 1.1950 | 0.0000 |
