# Supplementary material for: Human-specific mutations in VMAT1 confer functional changes and multi-directional evolution in the regulation of monoamine circuits
Source: BMC Evol Biol. 2019 Dec 2;19:220. doi: 10.1186/s12862-019-1543-8 (PMC6889191; doi:10.1186/s12862-019-1543-8)
Supplement: Supplementary file 7 — Additional file 7: Table S5. Primers used in the present study. [file 12862_2019_1543_MOESM7_ESM.docx]

**Table S5**. Used primers for generating constructs and confirming sequences.

| Purpose | Primer name | Sequence (5' to 3') | Start site^1^ |
| --- | --- | --- | --- |
| Amplification of *VMAT1* cDNA | Forward | AAAGATCTCTCCGGACCATTCTGGATG |  |
|  | Reverse | TTGAATTCCTACTCCTCATGGTCAGGC |  |
|  |  |  |  |
| Site-directed mutagenesis | I136T_F | GTTTCTTGGAGGAAGAGACTACCCGGGTCGGGGTTC |  |
|  | I136T_R | GAACCCCGACCCGGGTAGTCTCTTCCTCCAAGAAAC |  |
|  | I136N_F | GTTTCTTGGAGGAAGAGAATACCCGGGTCGGGGTTC |  |
|  | I136N_R | GAACCCCGACCCGGGTATTCTCTTCCTCCAAGAAAC |  |
|  | G130E_F | CTGCTTGCAAGGCACAGAATTCTTGGAGGAAGAGAA |  |
|  | G130E_R | TTCTCTTCCTCCAAGAATTCTGTGCCTTGCAAGCAG |  |
|  | N136T_F^2^ | GAATTCTTGGAGGAAGAGACTACCCGGGTCGGG |  |
|  | N136T_R^2^ | CCCGACCCGGGTAGTCTCTTCCTCCAAGAATTC |  |
|  |  |  |  |
| Sequence confirmation | F1 | GGCACCAAAATCAACGGGAC | –148 |
|  | F2 | AGGACGACGGCAACTACAAG | 304 |
|  | F3 | TCCGGACTCAGATCTCTCCG | 717 |
|  | F4 | CCGTGGCTGTTGAAGAAAGC | 994 |
|  | F5 | GGAGCTCCCTTTGGAAGTGT | 1455 |
|  | F6 | CCTCATTGGCACCAACCTCT | 1775 |

^1^The first nucleotide site of YFP was regarded as 0 and the last nucleotide of VMAT1 was the 2306^th^ site.

^2^These primers were used for the VMAT1 plasmid encoding 130Glu/136Asn.
